# The Western Journal of Medicine and Surgery

**Published:** 1840-02-15

**Authors:** 


### BIBLIOGRAPHICAL NOTICE.

The Western Journal of Medicine and Surgery: *Edited by* Daniel Drake, M. D., *and* Lunsford P. Yandell, M. D., *Professors in the Louisville Medical Institute.* No. 1. January, 1840.

The Western Journal of Medicine and Surgery, fills the void which has for a short time existed in the periodical medical literature of the west. It is intended more particularly to take the place of Dr. Drake's Cincinnati Journal, and of the Louisville Journal of Medicine and Surgery, both of which were lately suspended. The number, just received, is the first of a monthly series, and contains eighty-six octavo pages, neatly got up. Its prospects of success are, we think, good, occupying, as it does, the western field without competition; while from the reputation of the editors, and the merit of the number now issued, we have the promise of a useful and valuable addition to the corps of journals. It is but fair to mention that, although the character of the present number is highly creditable to the editors, they are unwilling it should be taken as a specimen of future numbers, which they feel confident of their ability to improve under more favourable circumstances.

We perceive that the new journal has enlisted the aid of a lively and powerful pen, with whose vigorous performances, if we mistake not, the profession has been long familiar. To this source we are now indebted for an able review of the lectures of the late Professor Hosack, published here about a year since. In the course of strictures upon this work, a question of much general interest is discussed, and an opinion advanced upon a point of medical education, to which we cannot altogether subscribe. The reviewer takes the ground that *practical* medicine cannot be taught to western pupils except by writers or lecturers of their own section of country. The mode of practice recommended in Dr. Hosack's work he considers utterly unsound, as respects the complaints of the Western States. "Nothing could tempt him to teach or recommend it to western students of medicine, and he would deeply grieve to see a sick friend treated in conformity to it." He is hence strengthened in the conviction that "the professors of the Atlantic schools are not qualified at all to communicate to their pupils such views of practical medicine as are suited to the diseases of the Mississippi valley."

In expressing our dissent from these views, we have no wish to give any particular or local bearing to our remarks. We have no doubt that western pupils may be perfectly well educated in every branch of medicine at Louisville, and other western schools, and feel satisfied that they will in general resort thither. But if the doctrine be carried out, that a student is to study medicine just in the spot where he is to practice it, our friends at Louisville will be narrowed down to a much more limited range of pupils than the "valley of the Mississippi." We apprehend that the same variety may be found between the diseases of different points of this locality as between many of them and those of the seaboard. A man of intelligence, who is thoroughly grounded in the elements of medicine, will find no difficulty in adapting general principles to peculiar circumstances. Practice, and practice only, will familiarize him with the characters of the epidemics which he is to encounter,—and these will vary with the varieties, not only of climate and latitude, but of years and seasons.

Besides, speaking comprehensively, the diseases of the middle States do not differ from those of the southern and western, except in degree; and a sound, judicious practice, is equally applicable to both. This is not a mere theoretical opinion; we have the direct testimony of fellow students, who have been for some years in practice, and of pupils who have entered upon practice within the last few years. Their testimony extends to every one of the southern and south western States, and to nearly all of those north of the Ohio river. There are, undoubtedly, many more cases, of what is called, in America, congestive fever, but is more generally known by the name of malignant and pernicious intermittent. But the diseases with us are the same as in the South, though generally of a much less degree in character.

We shall terminate these remarks with an extract from a letter from the middle of Tennessee, which alludes to congestive fever. "This," says the author, an accomplished physician, well known to one of the Editors of the Louisville Journal, is "nothing more than a malignant grade of intermittent. There is much in it calculated to alarm. It requires close watching, and a physician in this region, where his practice includes a large scope of country, loses a case occasionally, because he cannot give it the close attention required. One thing I have observed here, our pleurisies are more violent than yours; it is rare to meet with a case uncomplicated with pneumonia from the commencement."

In all parts of the United States, where fevers of the intermittent and remittent kind exist, these diseases are sufficiently alike for study.

